# Association of high-sensitivity C-reactive protein and anemia with acute kidney injury in neonates

**DOI:** 10.3389/fped.2022.882739

**Published:** 2022-11-02

**Authors:** Peiyao Zhang, Yuanyuan Tong, Deshan Yuan, Yixuan Li, Yu Jin, Liting Bai, Peng Gao, Wenting Wang, Jinxiao Hu, Xin Duan, Jinping Liu

**Affiliations:** ^1^ State Key Laboratory of Cardiovascular Disease, National Center for Cardiovascular Diseases, Fuwai Hospital, Chinese Academy of Medical Sciences and Peking Union Medical College, Beijing, China; ^2^ Department of Cardiopulmonary Bypass, Fuwai Hospital, Chinese Academy of Medical Sciences and Peking Union Medical College, Beijing, China; ^3^ Department of Anesthesiology, Beijing Tiantan Hospital, Capital Medical University, Beijing, China

**Keywords:** neonates, acute kidney injury, high-sensitivity C-reactive protein, anemia, congenital heart disease

## Abstract

**Background:**

The association of high-sensitivity C-reactive protein (hsCRP) and anemia with postoperative acute kidney injury (AKI) in neonates with congenital heart disease (CHD) is still unclear. The purpose of this study was to examine whether anemia-associated AKI is modulated by hsCRP in neonates.

**Methods:**

This study included 253 consecutive neonatal patients who underwent CHD surgery in a national tertiary hospital. We investigated the association between postoperative AKI with baseline hsCRP, anemia, and their interaction by multivariable logistic regression analyses.

**Results:**

The incidence of AKI was 24.1% in the entire cohort. After being adjusted for covariates, hsCRP level was negatively correlated with AKI (*P* < 0.01 for 1 mg/L threshold), whereas anemia emerged as an independent risk factor of AKI (*P* = 0.02). In addition, there was a significant interaction between anemia and hsCRP level (*P* = 0.01). In neonates with hsCRP < 1 mg/L, anemia was positively associated with AKI (*P* = 0.03). However, no significant association was found between anemia and AKI in the context of hsCRP ≥ 1 mg/L. Combination of anemia and hsCRP < 1 mg/L was independently correlated with the risk of AKI (*P* < 0.01), while concomitant anemia and hsCRP ≥ 1 mg/L or hsCRP < 1 mg/L combined with non-anemia was not.

**Conclusions:**

In neonates with CHD, the risk of anemia-associated AKI may be modulated by hsCRP level. Attention should be paid to neonates with preoperative anemia and baseline hsCRP < 1 mg/L to reduce the risk of postoperative AKI.

## Introduction

Early repair in the neonatal period or young infancy has become the treatment target of most patients with biventricular congenital heart disease (CHD) (1). Despite advances in neonatal CHD surgery, postoperative acute kidney injury (AKI) remains one of the leading complications that is independently correlated with morbidity and mortality (2, 3). Considerable risks despite symptomatic therapies have generated efforts to identify modifiable indicators and therapeutic measures for neonatal AKI (4, 5). Anemia is one putative target for mitigating the burden of AKI, according to authoritative data in adult cardiac surgery (6–8). However, the correlation between preoperative anemia and postoperative AKI has not been investigated in neonates, and it remains unclear which group of neonates would benefit the most from the elimination of anemia.

Systemic inflammation, which is caused by cardiovascular malformations and hypoperfusion of vital organs, has emerged as another potential pathogenesis exacerbating renal impairment (9–13). The interrelationship between inflammatory response and anemia may have a sophisticated effect on postoperative AKI. Among all inflammatory mediators, high-sensitivity C-reactive protein (hsCRP) has been demonstrated incontrovertibly as the most extensive predictive value for postoperative AKI in the context of adult cardiac surgery or cardiovascular intervention therapy (9–11, 14). Yet the consistency of its predictive role in adults and neonates is disputable (15). This study aimed to explore the hypothesis that a synergism between anemia and hsCRP on AKI in neonates undergoing CHD surgery, and whether anemia-associated AKI would be modulated by the degree of hsCRP, relying on the large-scale national tertiary hospital.

## Materials and methods

### Study population and data collection

We conducted a single-center retrospective study on neonatal patients (≤28 days) who underwent pump CHD primary repair from 1 November 2012 to 28 February 2021 at Fuwai Hospital, Chinese Academy of Medical Sciences. Preterm infants and neonates with preoperative extracorporeal membrane oxygenation support or emergency operation were excluded. This study was approved by the Institutional Review Board of Fuwai Hospital and performed with a waiver of informed consent from guardians in view of the retrospective nature.

Demographic characteristics, echocardiographic examination, predominant diagnosis, laboratory tests, intraoperative management, and postoperative information were collected. Based on the definition that concentration of hemoglobin (Hb) >2 standard deviations (SD) below the average of postnatal age (16) and the threshold of consideration for blood transfusion for neonates in our center, neonatal clinically relevant anemia was defined as Hb < 120 g/L. Serum hsCRP was routinely examined 1 day before operation by the clinical laboratory on Cobas c501 (Roche Diagnostics GmbH, Mannheim, Germany).

### Intraoperative management

The standard cardiopulmonary bypass (CPB) was established following systemic heparinization and median sternotomy. Red blood cell was generally not transfused before surgery. CPB circuit was primed with Plasmalyte A 40 ml, albumin 30 ml, packed red blood cell 80 ml, and sodium bicarbonate 10 ml. Cold histidine-tryptophan-ketoglutarate cardioplegia was perfused as body temperature cooled down to 27–30 °C. Hematocrit (Hct) was targeted at 0.24–0.27 during bypass. After lesions are repaired, neonates would be weaned from CPB only when hemodynamic state and body temperature reached satisfactory levels as sinus rhythm returned to normal. *α*-stat management was adopted during CPB. Hct was maintained at 0.35–0.40 by modified ultrafiltration. All neonates were transferred to the pediatric intensive care unit (PICU) after the procedure.

### AKI assessment

The primary outcome of this study was AKI. Neonates developing AKI at POD1 and still diagnosed with AKI 1 week after the operation were defined as having AKI within 1 week of surgery (17). AKI was determined by neonatal modified-Kidney Disease: Improving Global Outcomes (nKDIGO) criteria regardless of perioperative urine volume (18). AKI was detailed as follows: serum creatinine (SCr) increases of ≥26.5 *μ*mol/L within 48 h or ≥1.5 times the baseline value within 1 week or need for renal replacement therapy. Preoperative SCr rather than estimated glomerular filtration rate was employed in AKI diagnosis because the reliability of its formula has not been proven in neonates. Secondary outcomes included prolonged postoperative hospital stay (≥21 days), prolonged PICU stay (≥7 days), and delayed extubation (≥72 h). We also investigated the relationship between hepatic dysfunction and hsCRP level to explore the underlying cause of neonatal stress disorder. Hepatic dysfunction was defined as a total bilirubin level >2.5 mg/dl or >2-fold increase of alanine aminotransferase or aspartate aminotransferase from the baseline level (19).

### Statistical analysis

Continuous variables were presented as mean ± SD for normal distribution and median [interquartile range (IQR)] for non-normal distribution and were compared using independent-sample Student's *t*-test or Mann–Whitney *U* test, respectively. Categorical variables were reported as frequency (percentage) and were compared by Pearson *χ*^2^ test or Fisher's exact test when appropriate. Logistic regression models were generated to evaluate the association between preoperative hsCRP, anemia, and AKI, as well as interaction test, by adjusting for demographic characteristics, baseline laboratory tests, and intraoperative variables. The risk of AKI was compared between different combinations of hsCRP level and anemia. Two-tailed *P* < 0.05 was considered statistically significant. Statistical analysis was performed using SPSS 25.0 software (SPSS Inc., Chicago, IL, United States), and the figure was processed by GraphPad prism 8.0 (GraphPad Software Inc., San Diego, CA, United States).

## Results

### Baseline characteristics and perioperative information

A total of 262 consecutive neonates were identified, and 253 patients with complete medical records were finally included in this analysis. Baseline characteristics of the study population were classified by the presence (*n* = 61) or absence (*n* = 192) of AKI within 1 week of surgery (Table 1). Of the 253 neonates, 65.6% were male. The mean value of Hb and median of hsCRP were 142.82 g/L and 0.84 mg/L respectively. Neonates with AKI had a higher prevalence of anemia, compared with those without AKI (*P* = 0.03). Baseline hsCRP concentration was significantly lower in patients with AKI than those without (*P* < 0.01). For postoperative outcomes, there was a remarkably higher prevalence of prolonged PICU stay (≥7 days) among neonates with AKI (*P* = 0.01).

**Table 1:** Patient characteristics and perioperative information.

Variables	Overall (*n* = 253)	AKI within 1 week of surgery		*P*-value
		Non-AKI (*n* = 192)	AKI (*n* = 61)	
Baseline characteristics				
Male sex, *n* (%)	166 (65.6)	123 (64.1)	43 (70.5)	0.36
Age, day	15 (10, 21)	15 (9, 21)	16 (11, 23)	0.24
Weight, kg	3.60 (3.20, 3.90)	3.60 (3.20, 3.88)	3.60 (3.30, 3.90)	0.50
Body length, cm	50.00 (49.00, 52.00)	50.00 (49.00, 52.00)	51.00 (48.00, 53.00)	0.56
BSA, m^2^	0.22 (0.21, 0.23)	0.22 (0.20, 0.23)	0.22 (0.21, 0.23)	0.52
STAT score	3 (3, 4)	3 (3, 3)	3 (3,4)	0.05
LVEF, %	65.00 (63.00, 70.00)	65.00 (63.00, 70.00)	65.00 (60.00, 70.00)	0.26
Anemia, *n* (%)	53 (20.9)	34 (17.7)	19 (31.1)	0.03
Predominant diagnosis				
TGA, *n* (%)	163 (64.4)	126 (65.6)	37 (60.7)	0.48
APVD, *n* (%)	49 (19.4)	39 (20.3)	10 (16.4)	0.66
CoA, *n* (%)	14 (5.5)	9 (4.7)	5 (8.2)	0.30
Other CHD, *n* (%)	27 (10.7)	18 (9.4)	9 (14.8)	0.24
Preoperative laboratory test				
Hemoglobin, g/L	142.82 ± 23.13	144.76 ± 23.24	136.74 ± 21.87	0.02
Hematocrit, %	42.68 ± 7.69	43.23 ± 8.12	40.93 ± 5.84	0.04
Albumin, g/L	34.55 (31.40, 37.73)	34.55 (31.38, 37.93)	34.75 (31.63, 37.33)	0.82
Creatinine, μmol/L	34.38 (25.04, 43.75)	35.81 (28.94, 46.62)	25.26 (19.53, 44.63)	<0.01
hsCRP, mg/L	0.84 (0.20, 1.95)	0.98 (0.29, 2.28)	0.38 (0.12, 0.99)	<0.01
Glucose, mmol/L	5.39 (4.34, 6.61)	5.56 (4.34, 6.64)	5.21 (4.26, 6.46)	0.48
Lactate, mmol/L	1.20 (0.90, 1.50)	1.20 (0.90, 1.50)	1.10 (0.90, 1.30)	0.02
Intraoperative variables				
CPB time, min	124.50 (104.00, 150.75)	123.00 (104.00, 148.00)	130.00 (103.50, 153.50)	0.49
ACC time, min	82.00 (63.00, 104.50)	82.00 (63.00, 102.00)	83.00 (63.25, 106.75)	0.58
MAP, mmHg	39.00 (34.00, 46.00)	38.00 (33.00, 46.00)	40.00 (35.00, 45.00)	0.15
Postoperative outcomes				
Prolonged postoperative hospital stay (≥21 days), *n* (%)	118 (46.6)	90 (46.9)	28 (45.9)	0.98
Prolonged PICU stay (≥ 7 days), *n* (%)	104 (41.1)	71 (37.0)	33 (54.1)	0.01
Delayed extubation (≥72 h), *n* (%)	107 (42.2)	75 (39.1)	32 (54.5)	0.07

AKI, acute kidney injury; BSA, body surface area; CHD, congenital heart disease; STAT score, Society of Thoracic Surgeons-European Association for Cardio-Thoracic Surgery (STS-EACTS) mortality score; LVEF, left ventricular ejection fraction; TGA, transposition of the great arteries; APVD, anomalous pulmonary venous drainage; CoA, coarctation of aorta; hsCRP, high-sensitivity C-reactive protein; CPB, cardiopulmonary bypass; ACC, aortic cross-clamping; MAP, mean arterial pressure; PICU, pediatric intensive care unit. Other CHD includes single atrial septal defect, atrial septal defect and ventricular septal defect, and interruption of the aortic arch.

**Figure 1:**
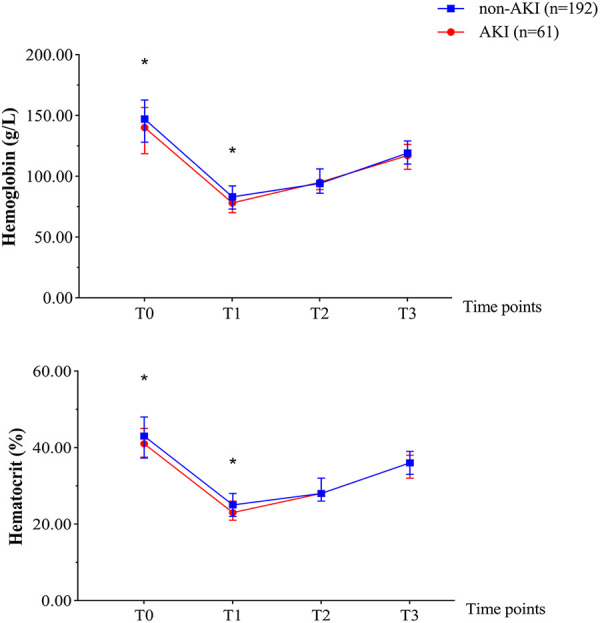
Perioperative hemoglobin and hematocrit of the two groups at different time points. T0, before surgery; T1, bypass hypothermia; T2, bypass rewarming; T3, weaning of bypass. *P < 0.05.

As Figure 1 illustrates, neonates with postoperative AKI had significantly lower Hb and Hct before the operation and during bypass hypothermia compared with those without AKI (*P* < 0.05).

### Association of hsCRP and anemia with AKI

Table 2 depicts the crude and multivariable-adjusted association between AKI and hsCRP or anemia. Baseline and intraoperative variables in Table 1 were submitted into multivariable analysis (forward stepwise regression). HsCRP elevation was defined as ≥1 mg/L in line with the cut-off value (hsCRP = 1.09 mg/L) of the receiver operating characteristic curve for AKI in this population (Supplementary Figure S1). As Table 2 presents, AKI was negatively associated with hsCRP assessed by threshold of 1 mg/L (odds ratio (OR): 0.34, 95% CI: 0.17–0.68, *P* < 0.01), and anemia was an independent risk factor for AKI after adjustment for cofounders (OR: 2.89, 95% CI: 1.23–6.79, *P* = 0.02).

**Table 2:** Association of hsCRP and anemia with AKI.

Variables	OR (95% CI)	*P*-value
Anemia vs. non-anemia		
Crude	2.10 (1.09–4.05)	0.03
Adjusted^a^	2.89 (1.23–6.79)	0.02
hsCRP ≥ 1 mg/L vs. hsCRP < 1 mg/L		
Crude	0.30 (0.15–0.58)	<0.01
Adjusted*^a^*	0.34 (0.17–0.68)	<0.01

hsCRP, high-sensitivity C-reactive protein; AKI, acute kidney injury.

^a^ Adjusted for gender, age, weight, body length, STAT score, baseline hematocrit, albumin, serum creatinine elevation (>40 μmol/L), hyperglycemia (>7 mmol/L), hyperlactacidemia (>2 mmol/L), and aortic cross-clamping time by forward: conditional.

### Association between AKI and anemia according to hsCRP threshold

As is revealed in Table 3, we stratified the entire cohort according to hsCRP threshold of 1 mg/L and found that anemia was correlated with a remarkably greater risk of AKI when hsCRP < 1 mg/L (OR: 2.67, 95% CI: 1.11–6.38, *P* = 0.03). Whereas in the setting of hsCRP ≥ 1 mg/L, anemia was not significantly associated with AKI. In addition, there was a significant interaction for AKI between anemia and hsCRP divided by the 1 mg/L threshold (*P* = 0.01).

**Table 3:** Risk of anemia-associated AKI according to hsCRP threshold.

Groups	OR (95% CI)	*P*-value	*P*-value for interaction*^a^*
hsCRP ≥ 1 mg/L			0.01
Non-anemia	1.00 (reference)	NA	
Anemia	1.47 (0.37–5.92)	0.59	
hsCRP < 1 mg/L			
Non-anemia	1.00 (reference)	NA	
Anemia	2.67 (1.11–6.38)	0.03^a^	

hsCRP, high-sensitivity C-reactive protein; AKI, acute kidney injury.

^a^ The same adjustment strategy as in Table 2.

### Risk of AKI in different combination groups of anemia and hsCRP

Table 4 indicates the results of the fully adjusted association between the risk of AKI and neonates classified by different combinations of anemia and hsCRP. Neither anemia combined with hsCRP ≥ 1 nor hsCRP < 1 mg/L without anemia was associated with a higher risk of AKI, compared with the reference group (non-anemia and hsCRP ≥ 1 mg/L). However, the combination of anemia and hsCRP < 1 mg/L significantly raised the risk of AKI (OR: 5.06, 95% CI: 1.85–13.82, *P* < 0.01).

**Table 4:** Risk of AKI stratified by anemia and hsCRP.

Groups	OR (95%CI)	*P*-value^a^
Non-anemia and hsCRP ≥ 1 mg/L	1.00 (reference)	NA
Anemia and hsCRP ≥ 1 mg/L	1.34 (0.32–5.63)	0.69
Non-anemia and hsCRP < 1 mg/L	2.25 (0.99–5.12)	0.06
Anemia and hsCRP < 1 mg/L	5.06 (1.85–13.82)	<0.01

hsCRP, high-sensitivity C-reactive protein; AKI, acute kidney injury.

^a^ The same adjustment strategy as in Table 2.

### Association between hsCRP and hepatic dysfunction

Table 5 shows that hsCRP < 1 mg/L was positively associated with postoperative hepatic dysfunction after being adjusted for cofounders (OR: 0.55, 95% CI: 0.30–0.99, *P* = 0.05).

**Table 5:** Association of hsCRP with hepatic dysfunction.

Hepatic dysfunction	OR (95% CI)	*P*-value
hsCRP ≥ 1 mg/L vs. hsCRP < 1 mg/L		
Crude	0.49 (0.28–0.83)	0.01
Adjusted^a^	0.55 (0.30–0.99)	0.05

hsCRP, high-sensitivity C-reactive protein.

^a^ The same adjustment strategy as in Table 2.

## Discussion

The core finding of this study was that the risk of anemia-associated AKI could be affected by the level of hsCRP in neonates undergoing CHD surgery. This is the first study, to our knowledge, that investigated the correlation of hsCRP level and anemia with adverse outcomes among neonates. Based on a relatively large neonatal cohort, we demonstrated that anemia was positively associated with AKI. Interestingly, neonates with concomitant existence of anemia and lower hsCRP level (<1 mg/L) had a higher risk of AKI, whereas neonates with concomitant anemia and hsCRP ≥ 1 or hsCRP < 1 mg/L combined with non-anemia did not, compared with those who had elevated hsCRP without anemia.

Given the prevalence of transient AKI in pediatric CHD surgery, AKI within 1 week of surgery was adopted as the primary endpoint of observation to avoid overdiagnosis. A recent study found that persistent AKI was strongly correlated with poorer in-hospital prognosis after CHD surgery (17). The remarkable risk of postoperative AKI remains despite advancement in the perioperative management of neonates (2, 4), constituting an impetus to the efforts for exploring modifiable risk factors. In the present study, we identified a 24.1% incidence of AKI consistent with previous studies (20, 21). As was reported in several infant studies (15, 22–25), lower preoperative SCr was the independent risk factor of cardiovascular surgery-associated AKI, especially for neonates. It corresponds with the results of this study. One possible explanation is that lower SCr in neonates may indicate less muscle mass and inferior nutritional status (15, 22–24), which may add to the risk of AKI. In addition, preoperative fluid overload may render a decrease in preoperative SCr level, which is also related to the higher risk of AKI (15). By adjusting for the factor of baseline SCr > 40 *μ*mol/L in multivariate logistic regression analyses, we eliminated the interference of latent renal insufficiency.

Compelling evidence in adults suggested that preoperative anemia was an independent predictor of AKI (6–8, 26). The major mechanisms underlying anemia-associated AKI involve renal oxygen supply impairment (16), exacerbation of oxidative stress (6), and hemostasis imbalance (7). This study provided consistent results that anemia was an independent risk factor for AKI in neonates undergoing cardiovascular surgery. However, we have noticed in clinical practice that postoperative AKI remained common in neonates even after anemia was corrected. Therefore, with the promotion of blood-saving strategy in infant CHD surgery (27) and the recognition of intraoperative blood transfusion-related risks (7), identifying the high-risk population of anemia-related AKI according to novel indicators is of vital importance to improve the prognosis of neonates as well as optimize the rational use of blood products.

The predictive value of baseline hsCRP for AKI has also been widely proposed in adult populations (9–12, 28). Previous adult studies identified hsCRP elevation as an independent biomarker of AKI in settings of percutaneous coronary intervention (10, 11) and coronary artery bypass grafting (9). Interestingly, the present study indicated a contrary to these results in adult studies, that in neonates undergoing CHD surgery, hsCRP elevation was negatively correlated with postoperative AKI.

In the context of hypoxic stress, CRP, which is also known as acute phase protein, was secreted by hepatocytes. CRP plays a critical role in preventing the generation of superoxide by neutrophils and stimulating the synthesis of interleukin-1 receptor antagonists, thus exerting a net anti-inflammatory effect (29). However, *in vivo* study indicated that CRP acts as a mediator in the pathogenesis of AKI and that overexpression of CRP inhibits the regeneration of G1/S-dependent tubular epithelial cells (30). Therefore, excessively high or low expression of CRP in stress response may both have a detrimental impact.

Generally, neonates with CHD bear extensive stress response due to hypoxia and hypoperfusion of vital organs. Immature organ development is the most distinguished characteristic of neonates that differs from patients of other ages. Our results showed that hsCRP < 1 mg/L was significantly correlated with hepatic dysfunction. An underdeveloped hepatic function may weaken the adaptive acute phase response of neonates to hypoxic stress. Also, it was demonstrated that younger patients may have a more pronounced systemic inflammatory response to critical diseases (31), and those preoperative CRP concentrations were inversely associated with the age of infants (1). Accordingly, we assumed that in the acute phase response, neonates with stress disorder characterized by limited elevation of hsCRP may have a higher risk of adverse events. Additionally, children suffering from CHD may commonly have chronic sympathetic activation and increased release of angiotensin (32). These mechanisms synergistically prompt the redistribution of blood flow from the kidneys to the brain and/or heart, which may lead to simulated preoperative renal ischemia preconditioning. The elevation of hsCRP (≥1 mg/L) may be a reflection of the activation of the inflammatory pathway induced by renal ischemic preconditioning (33). A period of nonfatal ischemic preconditioning has been tentatively conceptualized as protection against ischemic AKI (34). Furthermore, CRP has been reported to exert some anti-inflammatory effects by stimulating the release of anti-inflammatory factors including IL-10 and IL-1ra (35). CRP could also recruit several complement inhibitors when activating the complement system (36). Consequently, the net effect of CRP *in vivo* tends to be weak anti-inflammation (37), which may be another underlying explanation for the negative association between hsCRP elevation and AKI in this study.

The interaction between inflammation and anemia has not been investigated in neonates. In adult cohorts with chronic kidney disease, scholars have found that CRP exacerbated anemia by inhibiting endogenous/exogenous erythropoietin (38, 39), which may contribute to a synergic effect between inflammation and anemia on unfavorable events (39). However, data in this study indicated a different pattern between the interaction of hsCRP and anemia in neonates, that anemia was correlated with AKI only when hsCRP < 1 mg/L while this correlation was not valid in neonates with hsCRP ≥ 1 mg/L. Briefly, the risk of anemia-associated AKI in neonates could be mediated by hsCRP threshold. Therefore, what this study offered was the potential for refining the risk of AKI by identifying neonates with concomitant existence of hsCRP < 1 mg/L (may represent underlying stress disorder) and anemia.

## Limitations

There were certain limitations in this study. First, this study had all the inherent limitations of a retrospective study. Second, the single-center design limited the intensity of evidence though the sample size was relatively large as a neonate cohort. Third, the existing data could not confirm the causal relationship between hsCRP or anemia and adverse outcomes. Next, there was a lack of other inflammatory mediators in this study, which may otherwise help determine the degree of inflammation indicated by hsCRP. Furthermore, the incidence of AKI may be underestimated because we did not define AKI in terms of urine output. In addition, although preterm infants were excluded from the population in this study, gestational age may affect SCr and Hb. However, because our hospital is a specialized cardiovascular center and the neonatal patients were transported from other hospitals after birth, the data on gestational age in our database is incomplete to determine the effect of gestational age on the results. As there was a lack of SCr examination records more than 1 week after surgery for many neonates, we were unable to explore the AKI recovery since then retrospectively, which may exaggerate the correlation between hsCRP, anemia and AKI in this study. Therefore, corroboration of our conclusion needs to be verified in well-designed randomized controlled trials.

## Conclusions

Anemia-related AKI may be mediated by hsCRP level in the context of neonatal CHD surgery. This study offered a potential perspective that rectification of anemia in neonates, especially those combined with hsCRP < 1 mg/L may be beneficial for the improvement of clinical prognosis. Although the results of this study are interesting, it is only a preliminary study. The value of this study lies mainly in the use of limited data from a single center to emphasize the differences in the pathophysiology between neonates and older patients with CHD. We hope that this study could function as a starting point for multicenter studies to explore AKI prevention strategies or targets for the neonatal population specifically.

## Data Availability

The datasets presented in this article are not readily available because of data protection policies. Requests to access the datasets should be directed to the corresponding author.

## References

[1] Allan CK Newburger JW McGrath E Elder J Psoinos C Laussen PC The relationship between inflammatory activation and clinical outcome after infant cardiopulmonary bypass. Anesth Analg. (2010) 111(5):1244–51. 10.1213/ANE.0b013e3181f333aa 20829561

[2] Ueno K Shiokawa N Takahashi Y Nakae K Kawamura J Imoto Y Kidney disease: improving global outcomes in neonates with acute kidney injury after cardiac surgery. Clin Exp Nephrol. (2020) 24(2):167–73. 10.1007/s10157-019-01805-7 31677063

[3] Taylor ML Carmona F Thiagarajan RR Westgate L Ferguson MA del Nido PJ Mild postoperative acute kidney injury and outcomes after surgery for congenital heart disease. J Thorac Cardiovasc Surg. (2013) 146(1):146–52. 10.1016/j.jtcvs.2012.09.008 23040323

[4] Selewski DT Charlton JR Jetton JG Guillet R Mhanna MJ Askenazi DJ Neonatal acute kidney injury. Pediatrics. (2015) 136(2):e463–73. 10.1542/peds.2014-3819 26169430

[5] Alten JA Cooper DS Blinder JJ Selewski DT Tabbutt S Sasaki J Epidemiology of acute kidney injury after neonatal cardiac surgery: a report from the multicenter neonatal and pediatric heart and renal outcomes network. Crit Care Med. (2021) 49(10):e941–51. 10.1097/ccm.0000000000005165 34166288

[6] Karkouti K Yip P Chan C Chawla L Rao V. Pre-operative anaemia, intra-operative hepcidin concentration and acute kidney injury after cardiac surgery: a retrospective observational study. Anaesthesia (2018) 73(9):1097–102. 10.1111/anae.14274 29529338

[7] Karkouti K Grocott HP Hall R Jessen ME Kruger C Lerner AB Interrelationship of preoperative anemia, intraoperative anemia, and red blood cell transfusion as potentially modifiable risk factors for acute kidney injury in cardiac surgery: a historical multicentre cohort study. Can J Anaesth. (2015) 62(4):377–84. 10.1007/s12630-014-0302-y 25537735

[8] Karkouti K Wijeysundera DN Yau TM Callum JL Cheng DC Crowther M Acute kidney injury after cardiac surgery: focus on modifiable risk factors. Circulation. (2009) 119(4):495–502. 10.1161/CIRCULATIONAHA.108.786913 19153273

[9] Han SS Kim DK Kim S Chin HJ Chae DW Na KY. C-reactive protein predicts acute kidney injury and death after coronary artery bypass grafting. Ann Thorac Surg. (2017) 104(3):804–10. 10.1016/j.athoracsur.2017.01.075 28433221

[10] Gao F Zhou YJ Zhu X Wang ZJ Yang SW Shen H. C-reactive protein and the risk of contrast-induced acute kidney injury in patients undergoing percutaneous coronary intervention. Am J Nephrol. (2011) 34(3):203–10. 10.1159/000329534 21791916

[11] Shacham Y Leshem-Rubinow E Steinvil A Keren G Roth A Arbel Y. High sensitive C-reactive protein and the risk of acute kidney injury among ST elevation myocardial infarction patients undergoing primary percutaneous intervention. Clin Exp Nephrol. (2015) 19(5):838–43. 10.1007/s10157-014-1071-1 25492251

[12] Fu EL Franko MA Obergfell A Dekker FW Gabrielsen A Jernberg T High-sensitivity C-reactive protein and the risk of chronic kidney disease progression or acute kidney injury in post-myocardial infarction patients. Am Heart J. (2019) 216:20–9. 10.1016/j.ahj.2019.06.019 31382219

[13] Smilowitz NR Kunichoff D Garshick M Shah B Pillinger M Hochman JS C-reactive protein and clinical outcomes in patients with COVID-19. Eur Heart J. (2021) 42(23):2270–9. 10.1093/eurheartj/ehaa1103 33448289 PMC7928982

[14] Ridker PM. From C-reactive protein to interleukin-6 to interleukin-1: moving upstream to identify novel targets for atheroprotection. Circ Res. (2016) 118(1):145–56. 10.1161/CIRCRESAHA.115.306656 26837745 PMC4793711

[15] Morgan CJ Zappitelli M Robertson CM Alton GY Sauve RS Joffe AR Risk factors for and outcomes of acute kidney injury in neonates undergoing complex cardiac surgery. J Pediatr. (2013) 162(1):120–7 e121. 10.1016/j.jpeds.2012.06.054 22878115

[16] Colombatti R Sainati L Trevisanuto D. Anemia and transfusion in the neonate. Semin Fetal Neonatal Med. (2016) 21(1):2–9. 10.1016/j.siny.2015.12.001 26732078

[17] Jia Y Luo Q Su Z Xiong C Wang H Li Y The incidence and risk factors for persistent acute kidney injury following total cavopulmonary connection surgery: a single-center retrospective analysis of 465 children. Front Pediatr. (2021) 9:566195. 10.3389/fped.2021.566195 34307242 PMC8292609

[18] Shalaby MA Sawan ZA Nawawi E Alsaedi S Al-Wassia H Kari JA. Incidence, risk factors, and outcome of neonatal acute kidney injury: a prospective cohort study. Pediatr Nephrol. (2018) 33(9):1617–24. 10.1007/s00467-018-3966-7 29869723

[19] Tong Y Liu J Zou L Feng Z Zhou C Lv R Perioperative outcomes of using different temperature management strategies on pediatric patients undergoing aortic arch surgery: a single-center, 8-year study. Front Pediatr. (2018) 6:356. 10.3389/fped.2018.00356 30542643 PMC6277883

[20] Piggott KD Liu A Monczka J Fakioglu H Narasimhulu SS Pourmoghadam K Inadequate preoperative nutrition might be associated with acute kidney injury and greater illness severity postoperatively. J Thorac Cardiovasc Surg. (2018) 155(5):2104–9. 10.1016/j.jtcvs.2017.12.080 29366566

[21] Che R Quadri MM Zhang A. The epidemiology and management of pediatric AKI in Asia. Semin Nephrol. (2020) 40(5):516–32. 10.1016/j.semnephrol.2020.08.008 33334465

[22] Li S Krawczeski CD Zappitelli M Devarajan P Thiessen-Philbrook H Coca SG Incidence, risk factors, and outcomes of acute kidney injury after pediatric cardiac surgery: a prospective multicenter study. Crit Care Med. (2011) 39(6):1493–9. 10.1097/CCM.0b013e31821201d3 21336114 PMC3286600

[23] Morgan CJ Gill PJ Lamand S Joffe AR. Peri-operative interventions, but not inflammatory mediators, increase risk of acute kidney injury after cardiac surgery: a prospective cohort study. Intensive Care Med. (2013) 39(5):934–41. 10.1007/s00134-013-2849-4 23417202

[24] Zappitelli M Moffett BS Hyderand A Goldstein SL. Acute kidney injury in non-critically ill children treated with aminoglycoside antibiotics in a tertiary healthcare centre: a retrospective cohort study. Nephrol Dial Transplant. (2011) 26(1):144–50. 10.1093/ndt/gfq375 20591815

[25] Zappitelli M Krawczeski CD Devarajan P Wang Z Sint K Thiessen-Philbrook H Early postoperative serum cystatin C predicts severe acute kidney injury following pediatric cardiac surgery. Kidney Int. (2011) 80(6):655–62. 10.1038/ki.2011.123 21525851 PMC3312809

[26] Padmanabhan H Siau K Curtis J Ng A Menon S Luckraz H Preoperative anemia and outcomes in cardiovascular surgery: systematic review and meta-analysis. Ann Thorac Surg. (2019) 108(6):1840–8. 10.1016/j.athoracsur.2019.04.108 31233718

[27] Boettcher W Dehmel F Redlin M Sinzobahamvya N Photiadis J. Cardiopulmonary bypass strategy to facilitate transfusion-free congenital heart surgery in neonates and infants. Thorac Cardiovasc Surg. (2020) 68(1):2–14. 10.1055/s-0039-1700529 31679152

[28] Cosentino N Genovese S Campodonico J Bonomi A Lucci C Milazzo V High-sensitivity C-reactive protein and acute kidney injury in patients with acute myocardial infarction: a prospective observational study. J Clin Med. (2019) 8(12):2192. 10.3390/jcm8122192 PMC6947188 31842300

[29] Gabay C Kushner I. Acute-phase proteins and other systemic responses to inflammation. N Engl J Med. (1999) 340(6):448–54. 10.1056/nejm199902113400607 9971870

[30] Tang Y Huang XR Lv J Chung AC Zhang Y Chen JZ C-reactive protein promotes acute kidney injury by impairing G1/S-dependent tubular epithelium cell regeneration. Clin Sci (Lond). (2014) 126(9):645–59. 10.1042/CS20130471 24206243

[31] Butler J Pathi VL Paton RD Logan RW MacArthur KJ Jamieson MP Acute-phase responses to cardiopulmonary bypass in children weighing less than 10 kilograms. Ann Thorac Surg. (1996) 62(2):538–42. 10.1016/0003-4975(96)00325-6 8694619

[32] Hirano D Miwa S Kakegawa D Umeda C Takemasa Y Tokunaga A Impact of acute kidney injury in patients prescribed angiotensin-converting enzyme inhibitors over the first two years of life. Pediatr Nephrol. (2021) 36(7):1907–14. 10.1007/s00467-021-04920-4 33462699

[33] Bonventre JV Yang L. Cellular pathophysiology of ischemic acute kidney injury. J Clin Invest. (2011) 121(11):4210–21. 10.1172/JCI45161 22045571 PMC3204829

[34] Thiele RH Isbelland JM Rosner MH. AKI associated with cardiac surgery. Clin J Am Soc Nephrol. (2015) 10(3):500–14. 10.2215/CJN.07830814 25376763 PMC4348689

[35] Del Giudice M Gangestad SW. Rethinking IL-6 and CRP: why they are more than inflammatory biomarkers, and why it matters. Brain Behav Immun. (2018) 70:61–75. 10.1016/j.bbi.2018.02.013 29499302

[36] Mihlan M Blom AM Kupreishvili K Lauer N Stelzner K Bergström F Monomeric C-reactive protein modulates classic complement activation on necrotic cells. FASEB J. (2011) 25(12):4198–210. 10.1096/fj.11-186460 21856781

[37] Eisenhardt SU Thiele JR Bannasch H Starkand GB Peter K. C-reactive protein: how conformational changes influence inflammatory properties. Cell Cycle. (2009) 8(23):3885–92. 10.4161/cc.8.23.10068 19887916

[38] Gunnell J Yeun JY Depner TA Kaysen GA. Acute-phase response predicts erythropoietin resistance in hemodialysis and peritoneal dialysis patients. Am J Kidney Dis. (1999) 33(1):63–72. 10.1016/s0272-6386(99)70259-3 9915269

[39] Evans M Bower H Cockburn E Jacobson SH Barany P Carrero JJ. Contemporary management of anaemia, erythropoietin resistance and cardiovascular risk in patients with advanced chronic kidney disease: a nationwide analysis. Clin Kidney J. (2020) 13(5):821–7. 10.1093/ckj/sfaa054 33123358 PMC7577763

