# Supplementary figures and images for: Association of high-sensitivity C-reactive protein and anemia with acute kidney injury in neonates
Source: Front Pediatr. 2022 Nov 2;10:882739. doi: 10.3389/fped.2022.882739 (PMC9666738; doi:10.3389/fped.2022.882739)

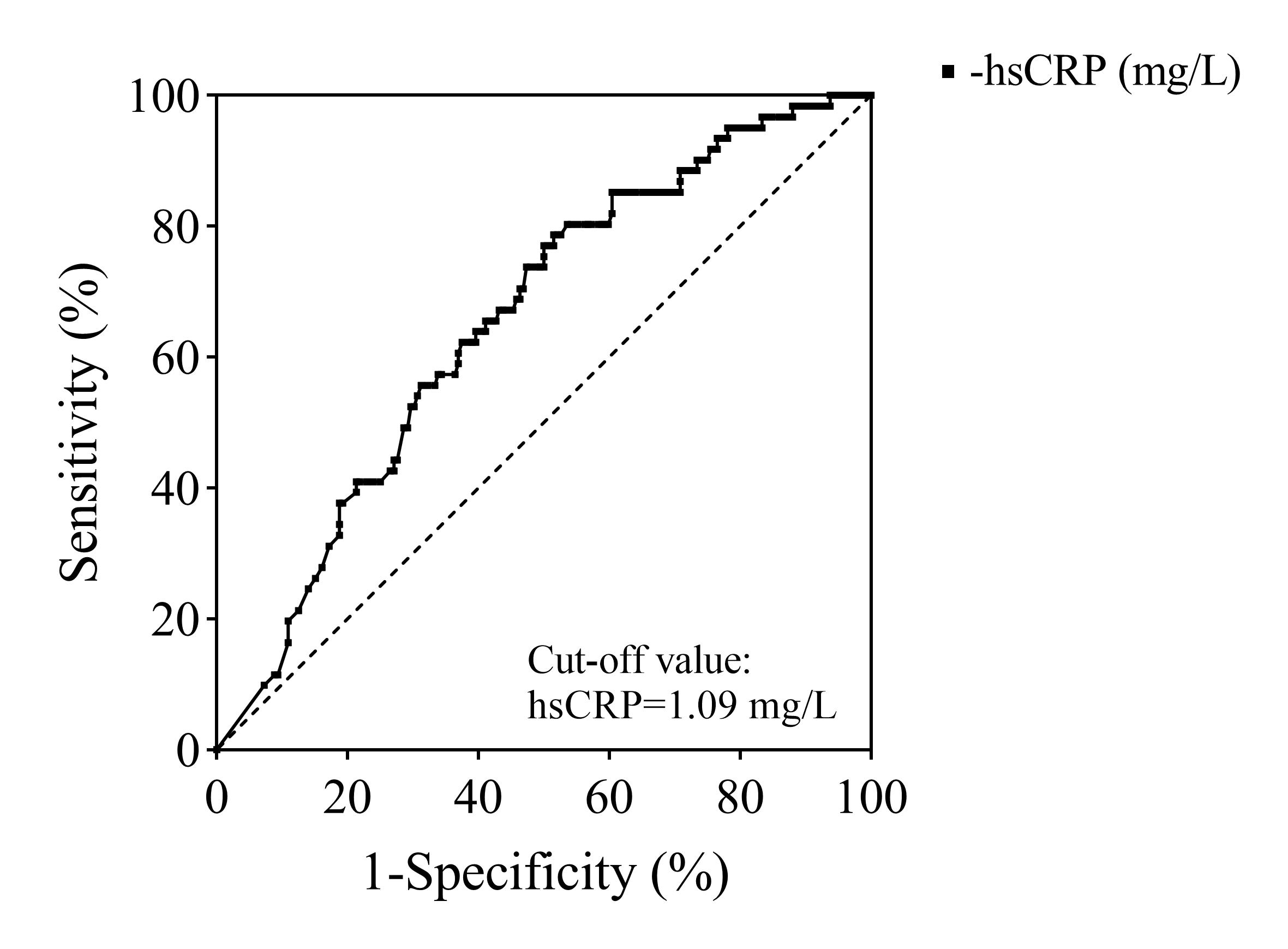

Supplement: Supplementary file 1 [file Image1.tif]
